# Design of a multi-epitope vaccine against goatpox virus using an immunoinformatics approach

**DOI:** 10.3389/fcimb.2023.1309096

**Published:** 2024-02-29

**Authors:** Qinqin Long, Min Wei, Yuting Wang, Feng Pang

**Affiliations:** Department of Veterinary Medicine, College of Animal Science, Guizhou University, Guiyang, China

**Keywords:** goatpox virus, epitope vaccine, immunoinformatics, molecular docking, molecular dynamics, immune simulation

## Abstract

**Introduction:**

Goatpox, a severe infectious disease caused by goatpox virus (GTPV), leads to enormous economic losses in the livestock industry. Traditional live attenuated vaccines cause serious side effects and exist a risk of dispersal. Therefore, it is urgent to develop efficient and safer vaccines to prevent and control of GTPV.

**Methods:**

In the present study, we are aimed to design a multi-epitope subunit vaccine against GTPV using an immunoinformatics approach. Various immunodominant cytotoxic T lymphocytes (CTL) epitopes, helper T lymphocytes (HTL) epitopes, and B-cell epitopes from P32, L1R, and 095 proteins of GTPV were screened and liked by the AAY, GPGPG, and KK connectors, respectively. Furthermore, an adjuvant β-defensin was attached to the vaccine’s N-terminal using the EAAAK linker to enhance immunogenicity.

**Results:**

The constructed vaccine was soluble, non-allergenic and non-toxic and exhibited high levels of antigenicity and immunogenicity. The vaccine’s 3D structure was subsequently predicted, refined and validated, resulting in an optimized model with a Z-value of -3.4. Molecular docking results demonstrated that the vaccine had strong binding affinity with TLR2(-27.25 kcal/mol), TLR3(-39.84 kcal/mol), and TLR4(-59.42 kcal/mol). Molecular dynamics simulation results indicated that docked vaccine-TLR complexes were stable. Immune simulation analysis suggested that the vaccine can induce remarkable increase in antibody titers of IgG and IgM, higher levels of IFN-γ and IL-2.

**Conclusion:**

The designed GTPV multi-epitope vaccine is structurally stable and can induce robust humoral and cellular immune responses, which may be a promising vaccine candidate against GTPV.

## 1 Introduction

Goatpox, caused by goatpox virus (GTPV), is a serious, highly contagious infectious disease that causes enormous economic losses in the goat industry (Tadesse et al., 2022). The typical clinical symptoms of goatpox are characterized by pyrexia, excessive salivation, widespread pock lesions on the skin, enlargement of superficial lymph nodes, and pulmonary lesions (Zhou et al., 2012; Zewdie et al., 2021). Goatpox is endemic in Africa, the Middle East, part of Europe and Asia including China mainland, which can cause very high morbidity and mortality even above 50% in young animals. Due to its severe economic impact and higher mortality, goatpox is listed as a notifiable transboundary animal disease by OIE (Zeng et al., 2014; Tuppurainen et al., 2017; Pham et al., 2021). GTPV, together with sheeppox virus (SPPV) and lumpy skin disease virus (LSDV), belong to the genus *Capripoxvirus* (CaPV) in the family *Poxviridae* (Dharanesha et al., 2020; Hamdi et al., 2021). Capripoxviruses are extremely conserved with 96% genome identity between SPPV, GTPV and LSDV(Tulman et al., 2002). The GTPV genome is a linear double-stranded DNA of approximately 150 kb in length, consisting of 156 putative open reading frames (ORFs). Among these, the conserved essential genes involved in viral replication and assembly lie in the central core region (ORFs024 to 123) while those responsible for pathogenicity and host range are located in the terminal variable region (ORFs01 to 023 and 124 to 156) (Santhamani et al., 2014; Sumana et al., 2020).

P32, a homologue of vaccinia virus (VACV) H3L, is a major immunodominant envelope protein present on the IMV surface of all capripoxviruses (Sumana et al., 2020). It has been reported that recombinant H3L protein induced high titers neutralizing antibodies and elicited protection against lethal challenge in mice (Davies et al., 2005). Recombinant P32 protein or its truncated products generated from multiple expression systems have been widely used as predominate antigens in ELISA for serological diagnosis of capripoxvirus infection in sheep and goats (Bhanot et al., 2009; Tian et al., 2010; Venkatesan et al., 2018). L1R is a myristoylated envelope protein present on the IMV surface of all capripoxviruses and plays a critical role in viral morphogenesis and virion entry. Sequence analysis of CaPV L1R gene revealed more than 96% identity within species and between species at both nucleotide and amino acid levels (Bisht et al., 2008; Karki et al., 2018). Zheng and colleagues demonstrated that A27L-L1R DNA vaccine elicited strong humoral and cellular immunity in mice and goats and provided partial protection against GTPV challenge in goats with reduced side reactions (Zheng et al., 2009). Hooper et al. found that vaccination with L1R gene alone evoked neutralizing antibody and the mice were partially protected. When vaccinated with a combination of both L1R and A33R gene induced more effective protection against lethal VACV challenge (Hooper et al., 2000). Furthermore, the mice were completely protected when vaccinated with a combination of four VACV genes (A27L+A33R+L1R +B5R) (Hooper et al., 2003). GTPV ORF095, a homologue of VACV A4L, was an immunodominant virion core protein synthesized at a later stage of infection and can induce strong humoral immune response (Kushwaha et al., 2019). Multiple sequence alignment analysis revealed that ORF095 gene was highly conserved among different CaPV isolates and it was predicted to be an ideal diagnostic antigen for the development of immunoassays (Madhavan et al., 2020).

GTPV and SPPV are generally considered as host-specific because the occurrence of outbreaks predominantly in either goats or sheep. However, they may sometimes cross the species barrier and infect each other. A few live attenuated vaccines have been developed and licensed for commercial use to combat GTPV infections in endemic areas. They are reported to provide long-lasting protective immunity if sufficient herd immunity is maintained by carrying out annual vaccination (Hosamani et al., 2004; Zhu et al., 2018; Bhanuprakash et al., 2022). However, the inoculation of live attenuated vaccines can cause serious side effects such as local pock lesions and miscarriage in pregnant goats. There also exists a risk that an attenuated vaccine virus can revert back to virulent state (Rao and Bandyopadhyay, 2000; Bhanuprakash et al., 2012). Therefore, it is urgent to develop efficient and safer vaccines to prevent and control of GTPV. Multi-epitope vaccines are developed using reverse vaccinology and immunoinformatics approaches by screening for pathogenic dominant epitopes (CTL epitopes, HTL epitopes and B-cell epitopes) and cascading them to obtain multi-epitope constructs (Aziz et al., 2022; Huang et al., 2022). Compared to experimental techniques, the development of a multi-epitope vaccine is more convenient, less expensive, and less time-consuming. Furthermore, the resulting multi-epitope vaccines have the following benefits of simultaneously inducing both cellular and humoral immunity against specific pathogens with minimal allergenicity and toxicity compared to conventional vaccines. Multi-epitope-based vaccine models are currently being constructed against a wide range of viruses, including SARS-Cov2, monkeypox virus, human herpes virus and Influenza virus (Dong et al., 2020; Jiang et al., 2023; Rcheulishvili et al., 2023; Suleman et al., 2023). However, a multi-epitope vaccine against GTPV has not been developed.

The aim of this study was to design a multi-epitope subunit vaccine against GTPV using reverse vaccinology and immunoinformatics approaches. First, the main immunogenic proteins P32, L1R and 095 of GTPV were used as the target antigens, and their dominant cytotoxic T-cell (CTL), helper T-cell (HTL) and B-cell epitopes were screened and tandemly linked by flexible junctions to obtain the GTPV multi-epitope vaccine. Then, the physicochemical properties, antigenicity, immunogenicity, allergenicity, toxicity, secondary and 3D structure of the constructed multi-epitope vaccine were predicted. Furthermore, we performed molecular docking, molecular dynamic simulation, immune simulation, codon optimization and *in silico* cloning to evaluate its characteristics and potential protective efficacy of the constructed multi-epitope vaccine.

## 2 Methods

### 2.1 Protein sequence retrieval

The entire amino acid sequences of P32 (YP_001293265.1), L1R (ABS72327.1) and 095 (YP_001293290.1) protein of GTPV in FASTA format were retrieved from NCBI database (https://www.ncbi.nlm.nih.gov/).

### 2.2 Screening of cytotoxic T lymphocytes epitopes

Cytotoxic T lymphocytes (CTLs) that are activated by MHC-I epitopes (CTL epitopes) can directly eradicate viruses and infected cells from the host, thereby playing a critical role in cellular immunity (Aiman et al., 2022). The MHC-I binding server (https://tools.iedb.org/mhci/) in IEDB was used to predict CTL epitopes(9 mer) of selected antigens (Andreatta and Nielsen, 2016). The prediction method was NetMHCpan 4.1 EL and the selected alleles were human HLA-A^*^01:01 and HLA-A^*^02:01. Epitopes with percentile rank<0.5 were retained for further analysis. The VaxiJen v2.0 server (https://www.ddg-pharmfac.net/vaxijen/VaxiJen/VaxiJen.html) was then used to predict the antigenicity of selected epitopes with a threshold value of 0.4 (Doytchinova and Flower, 2007). Finally, the immune-enhancing epitopes were identified as qualified CTL epitopes for the multi-epitope vaccine construction.

### 2.3 Screening of helper T lymphocyte epitopes

Helper-T cells (HTLs) are involved in activating both the B-cell and cytotoxic T-cell pathways, can assist both humoral and cellular immunity (Hou et al., 2023). The MHC-II binding server (https://tools.iedb.org/mhcii/) in IEDB was utilized to predict the HTL epitopes (15 mer) of selected antigens. The prediction method was NetMHCIIpan 4.1 EL and the selected alleles were human DRB reference set (DRB1^*^01:01, DRB1^*^01:02) (Jensen et al., 2018). A percentile rank<0.5 was set as the filtering standard for HTL epitopes candidates. The antigenicity of these candidate epitopes was subsequently evaluated using VaxiJen v2.0 with a threshold of 0.4 (Doytchinova and Flower, 2007). The IFN-γ epitope server (https://crdd.osdd.net/raghAVA/ifnepitope/index.php) was also used to evaluate the interferon gamma (IFN-γ) inducibility of selected epitopes (Dhanda et al., 2013). Only HTL epitopes with a positive IFN-γ score were retained. These final epitopes meeting the above criteria were selected as qualified HTL epitopes for the multi-epitope vaccine construction.

### 2.4 Screening of linear B-cell epitopes

B-cell epitopes are essential components of multi-epitope vaccine since they play a significant role in humoral immunity by generating specific neutralizing antibodies to protect hosts against invading pathogens (Moin et al., 2022). The ABCpred server (https://webs.iiitd.edu.in/raghava/abcpred/index.html) was utilized for the prediction and screening of immunodominant linear B-cell epitopes due to its high accuracy (Saha and Raghava, 2006; Saha and Raghava, 2007). The filtering threshold was kept at 0.85 and the length of epitopes was limited to 16.

### 2.5 Construction of the multi-epitope vaccine

A GTPV multi-epitope vaccine was constructed by merging the screened immunodominant CTL, HTL and B-cell epitopes by AAY, GPGPG and KK linkers, respectively. To increase immunogenicity, an adjuvant β-defensin-3 (GenBank: AAV41025.1) was attached to the vaccine’s N-terminal using the EAAAK linker.

### 2.6 Assessment of physicochemical properties, solubility, antigenicity, immunogenicity, allergenicity and toxicity of the multi-epitope vaccine

The Expasy Protparam server (https://web.expasy.org/protparam/) was utilized to predict the physicochemical properties of the multi-epitope vaccine (Wilkins et al., 1999). The Protein-Sol Server (https://protein-sol.manchester.ac.uk/) was used to predict the solubility of the designed multi-epitope vaccine (Hebditch et al., 2017). The ANTIGENpro server (https://scratch.proteomics.ics.uci.edu/) (Magnan et al., 2010) and IEDB Immunogenicity server (http://tools.iedb.org/immunogenicity/) (Calis et al., 2013) were exploited to predict the antigenicity and immunogenicity of the constructed vaccine. The allergenicity and toxicity of the vaccine were subsequently predicted using AllerScreener (https://www.ddg-pharmfac.net/AllerScreener/screen/) and ToxinPred server, respectively (https://crdd.osdd.net/raghava/toxinpred/) (Gupta et al., 2013).

### 2.7 Modeling, refinement and validation of the multi-epitope vaccine

Secondary structure of the constructed multi-epitope vaccine was predicted by the PSIPRED 4.0 server (http://bioinf.cs.ucl.ac.uk/psipred/) (Buchan and Jones, 2019). Next, the 3D structure of the multi-epitope vaccine was predicted by Phyre2 server (http://www.sbg.bio.ic.ac.uk/phyre2/html/page.cgi?id=index) (Wass et al., 2010; Kelley et al., 2015). The 3D model was then refined using GalaxyRefine server (https://galaxy.seoklab.org/cgi-bin/submit.cgi?type=REFINE) (Ko et al., 2012; Heo et al., 2016). The finalized model was further validated via ProSA-Web (https://prosa.services.came.sbg.ac.at/prosa.php) (Sippl, 1993; Wiederstein and Sippl, 2007). Ramachandran plots showed dominant, anomalous and rotational isomer regions in the 3D structures before and after optimization using the SWISS-MODEL server (https://swissmodel.expasy.org/assess) (Bienert et al., 2017; Waterhouse et al., 2018).

### 2.8 Molecular docking

Molecular docking is used to study the binding affinity between vaccine candidates and immune receptors. PDB files for TLR2 (PDB ID:6NIG), TLR3 (PDB ID:2A0Z) and TLR4 (PDB ID:4G8A) were obtained from RCSB PDB database. Then, the protein-protein docking of GTPV-M vaccine with TLR2, TLR3 and TLR4 were performed through HawkDock web server (http://cadd.zju.edu.cn/hawkdock/) (Weng et al., 2019). The binding free energy of the best-docked model of vaccine-TLRs complex was further predicted via MM/GBSA (Molecular Mechanics/Generalized Born Surface Area) analysis. Besides, the PDBsum server (http://www.ebi.ac.uk/thornton-srv/databases/pdbsum/) was used to investigate the interacting residues between docked chains (Laskowski et al., 2018).

### 2.9 Molecular dynamic simulation

Molecular dynamics (MD) simulation was used to predict the stability of the vaccine-receptor complex with the best docking model using iMODS web server (https://imods.iqfr.csic.es/) (Lopez-Blanco et al., 2011; Lopez-Blanco et al., 2014). Normal Modal Analysis (NMA) was used to demonstrate the coordinated motion of the multi-epitope vaccine in internal coordinates. Furthermore, the following evaluation parameters consist of B-factor, deformability, covariance, variances, elastic model, and eigenvalues were implemented to understand the stability of the docked complex (Ahmad et al., 2020; Ullah et al., 2023).

### 2.10 Immune simulation

Computerized immune simulations were performed using the C-ImmSim server (https://kraken.iac.rm.cnr.it/C-IMMSIM/) to evaluate the immune response of the multi-epitope vaccine (Rapin et al., 2010). Three injections were set and the time interval between each dose was four weeks. Total simulation steps were set to 1050 (1-time step equals 8 h) and each time step of injection was 1, 84, and 168. respectively.

### 2.11 Codon optimization and in silico cloning

To optimize the expression rate of the designed vaccine in the *E.coli* expression system, the peptide sequence was submitted to the JCat web server (https://www.jcat.de/Start.jsp) for reverse translation and codon optimization in the *E.coli* K12 host strain (Grote et al., 2005). The GC content percentage and the codon adaptation index (CAI) were analyzed to evaluate the transcription and translation efficiency. A higher level of exogenous gene expression is indicated by a higher CAI value approaching 1 and an ideal GC content between 30% and 70% (Puigbo et al., 2008). Further, restriction sites for *Bam*HI and *Xho*I at the N- and C-terminals were added to enable restriction cloning into the pET-28a (+) vector using the SnapGene software.

## 3 Results

### 3.1 Screening of immunodominant CTL, HTL and B-cell epitopes

**Figure 1:**
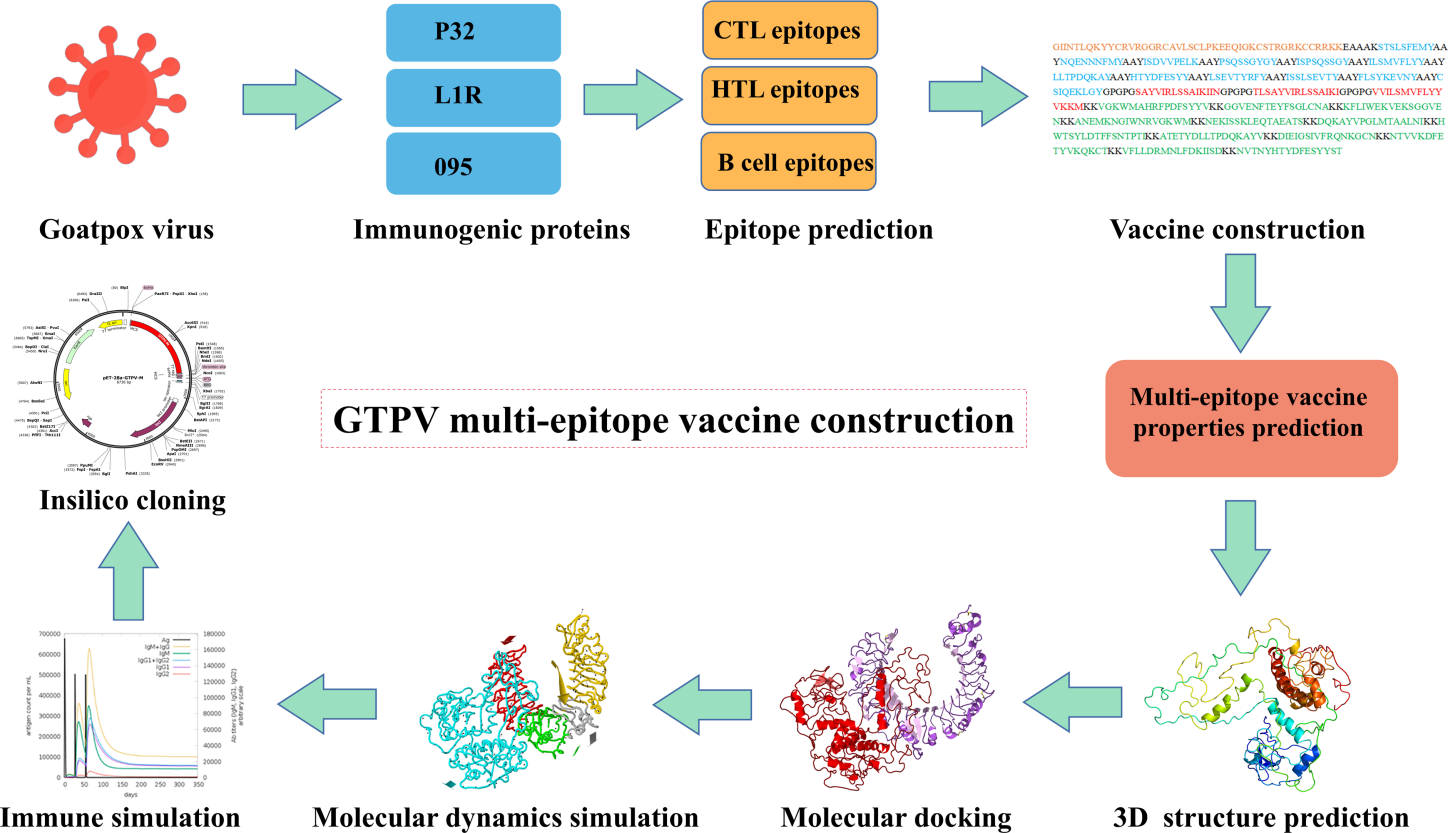
Flow chart for constructing the multi-epitope vaccine.

Three protein targets, P32, L1R, and 095 were selected for their good antigenicity and ability to induce neutralizing antibodies. We further screened their immunodominant B-cell epitopes and T-cell epitopes. Based on IEDB results with percentile rank less than 0.5 and antigenicity score greater than 0.4, a total of 12 immunodominant CTL epitopes from P32, L1R and 095 proteins of GTPV were screened (**Table 1**). Three immunodominant HTL epitopes were selected based on the following criteria: percentile rank was less than 0.5, antigenicity score was greater than 0.4, and IFN-γ inducer score was positive (**Table 1**). Twelve immunodominant B-cell epitopes with an ABCpred score of 0.85 or higher have been identified for the multi-epitope vaccine construction (**Table 2**). **Figure 1** shows a flow chart for the construction of the GTPV multi-epitope vaccine.

**Table 1:** Immunodominant CTL and HTL epitopes selected for vaccine design.

Protein	Epitope type	Epitope sequence	Position	Percentile rank	Antigenicity score	IFN-γ inducer score
P32	CTL	STSLSFEMY	207-215	0.04	1.3553	–
		NQENNNFMY	165-173	0.18	0.8446	–
		ISDVVPELK	16-24	0.33	1.1856	–
	HTL	SAYVIRLSSAIKIIN	184-198	0.30	0.4386	0.4033
		TLSAYVIRLSSAIKI	182-196	0.30	0.4539	0.5629
L1R	CTL	PSQSSGYGY	176-184	0.19	0.3969	–
		ISPSQSSGY	174-182	0.2	0.6396	–
		ILSMVFLYY	195-203	0.39	0.6350	–
		LLTPDQKAY	78-86	0.45	0.5799	–
	HTL	VVILSMVFLYYVKM	193-207	0.19	0.6548	0.0855
095	CTL	HTYDFESYY	151-159	0.04	0.4242	–
		LSEVTYRFY	188-196	0.06	0.9230	–
		ISSLSEVTY	185-193	0.09	0.8483	–
		FLSYKEVNY	65-73	0.34	1.7070	–
		CSIQEKLGY	137-145	0.36	1.2266	–

**Table 2:** Immunodominant B-cell epitopes selected for vaccine design.

Protein	Peptide sequence	Position	ABCpred score
P32	VGKWMAHRFPDFSYYV	258-273	0.94
	GGVENFTEYFSGLCNA	71-86	0.89
	KFLIWEKVEKSGGVEN	60-75	0.87
	ANEMKNGIWNRVGKWM	247-262	0.86
L1R	NEKISSKLEQTAEATS	15-20	0.93
	DQKAYVPGLMTAALNI	82-97	0.91
	HWTSYLDTFFSNTPTI	227-242	0.91
	ATETYDLLTPDQKAYV	72-87	0.90
	DIEIGSIVFRQNKGCN	35-50	0.88
	NTVVKDFETYVKQKCT	102-117	0.87
095	VFLLDRMNLFDKIISD	253-268	0.87
	NVTNYHTYDFESYYST	146-161	0.87

### 3.2 Multi-epitope vaccine construction

**Figure 2:**
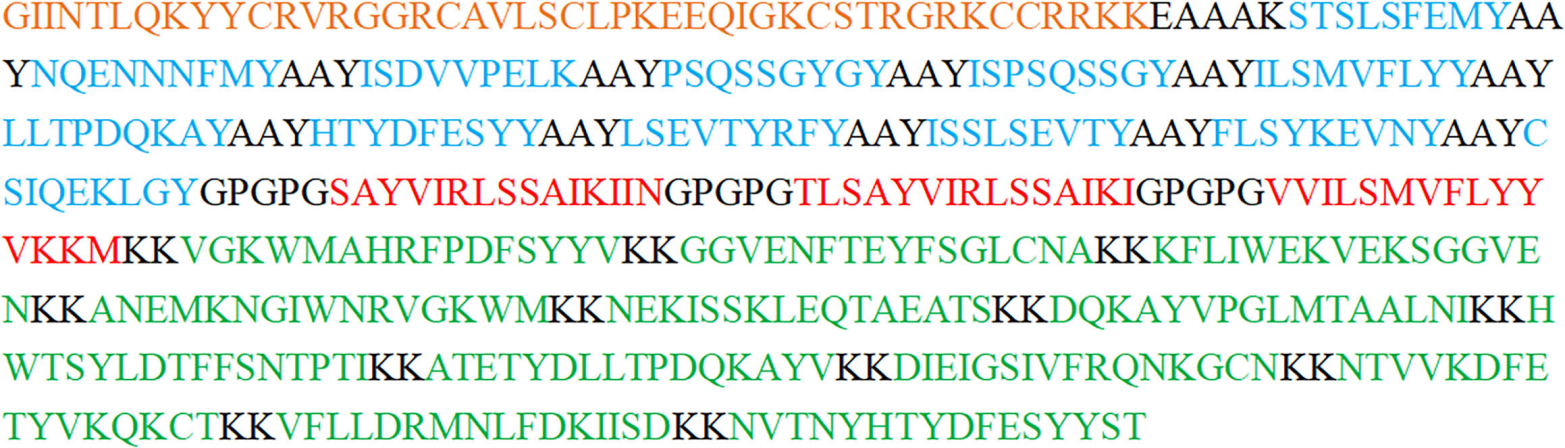
The construction of the GTPV-M vaccine. (Orange indicates β-defensin adjuvant; Blue indicates CTL epitopes; Red indicates HTL epitopes; Green indicates B-cell epitopes. Residues in black include the EAAAK connector between β-defensin adjuvant and CTL epitopes, the AAY connector between CTL epitopes, the GPGPG connector between HTL epitopes, and the KK connector between B-cell epitopes).

To develop a multi-epitope vaccine against GTPV, we combined all the screened 12 CTL epitopes, 3 HTL epitopes and 12 B-cell epitopes from P32, L1R and 095 protein using the AAY, GPGPG, and KK linkers, respectively. Furthermore, an adjuvant β-defensin was attached to the N-terminal of the vaccine using the EAAAK linker to enhance immunogenicity (**Figure 2**). Finally, a GTPV multi-epitope vaccine named GTPV-M was constructed.

### 3.3 Physiochemical properties, solubility, antigenicity, immunogenicity, allergenicity and toxicity of the multi-epitope vaccine

Expasy Protparam server results suggest that the constructed GTPV-M vaccine consists of 467 amino acids. The molecular weight of the vaccine is approximately 53.04 kDa and its theoretical PI is 9.44. The half-life of the vaccine in *E.coli* is estimated more than 10 hours and the instability index is 34.85 less than 40, which means the constructed GTPV-M vaccine is stable and easily expressed in prokaryotic expression system. The grand average of hydropathicity (GRAVY) of the vaccine is -0.368, demonstrating that it has a good hydrophilicity. The solubility value of the GTPV-M vaccine is 0.471 (>0.45) according to the results of Protein-Sol server, which indicates a good solubility. The antigenicity value of the GTPV-M vaccine is 0.81 and its immunogenicity value is -7.70, indicating that the GTPV-M vaccine has a high level of antigenicity and immunogenicity, enabling it to induce a strong immune response. Furthermore, the GTPV-M vaccine is non-allergenic and non-toxic based on results from AllerScreener and ToxinPred server.

### 3.4 Secondary structure of the multi-epitope vaccine

The secondary structure of the final construct was predicted by PSIPRED 4.0 server and the results suggested that the GTPV-M vaccine consists of 48.18% α-helix, 19.27% extended strand, 25.05% random coil and 7.49% β-turn, respectively (**Supplementary Figure 1**). Among them, α-helix, which is important for the structure and function of proteins, accounts for the highest proportion in the secondary structure of GTPV-M vaccine.

### 3.5 3D modeling, refinement and validation of the multi-epitope vaccine

**Figure 3:**
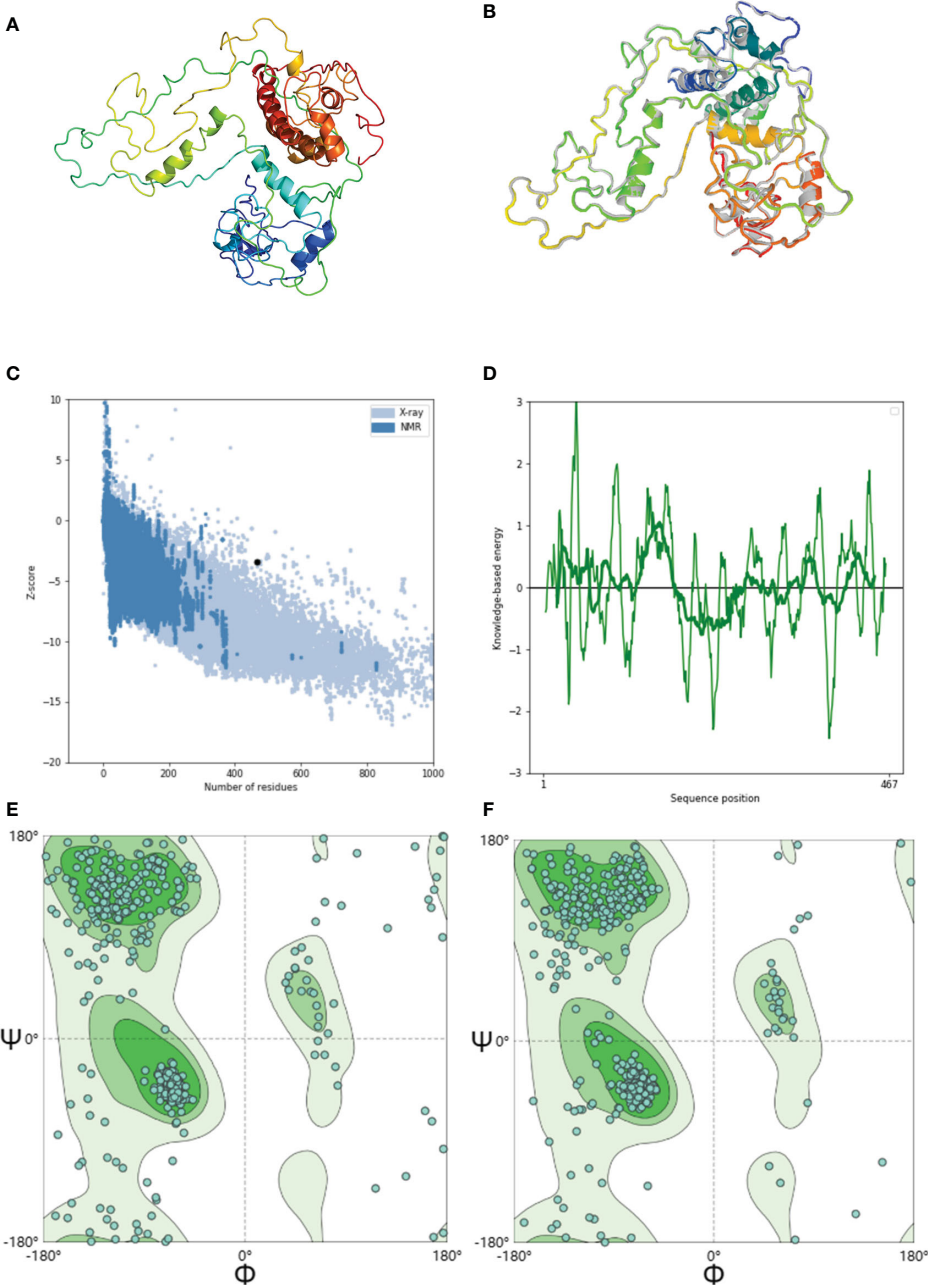
Modeling, refinement and validation of GTPV-M vaccine. **(A)**. Tertiary structure of GTPV-M vaccine; **(B)**. Optimized tertiary structure of GTPV-M vaccine; **(C)**. Z-score of the optimized model is -3.4; **(D)**. Energy map of the optimized model; **(E)**. Before optimization, the Ramachandran plot showed the dominant, anomalous and rotational isomer regions in GTPV-M vaccine were 77.63%, 6.67%, and 15.7%, respectively; **(F)**. After optimization, the Ramachandran plot showed the dominant, anomalous and rotational isomer regions in GTPV-M vaccine were 86.45%, 2.37% and 11.17%, respectively.

The 3D structure of the GTPV-M vaccine has been predicted by phyre2 online server (**Figure 3A**). The 3D model was further refined using the GalaxyRefine web server, and the quality and potential errors of the 3D model were checked and verified using the ProSA web server. The GalaxyRefine web server presents five optimized 3D models. Within the finest model (**Figure 3B**), the GDTHA value was 0.9363, the RMSD value was 0.467, the MolProbity value was 2.578, and the Clash score was 28.7. The ProSA web shows that the overall quality Z-value of the optimized model is -3.4, which is considered to be of higher quality (**Figure 3C**). The energy plot is shown in **Figure 3D**. SWISS-MODEL server evaluation found that the original model had 77.63% dominant region, 6.67% anomalous region and 15.7% rotational isomer region as shown in **Figure 3E**. After optimization, the dominant, anomalous and rotational isomer regions are 86.45%, 2.37% and 11.17%, respectively (**Figure 3F**).

### 3.6 Molecular docking between GTPV-M vaccine with TLRs

**Figure 4:**
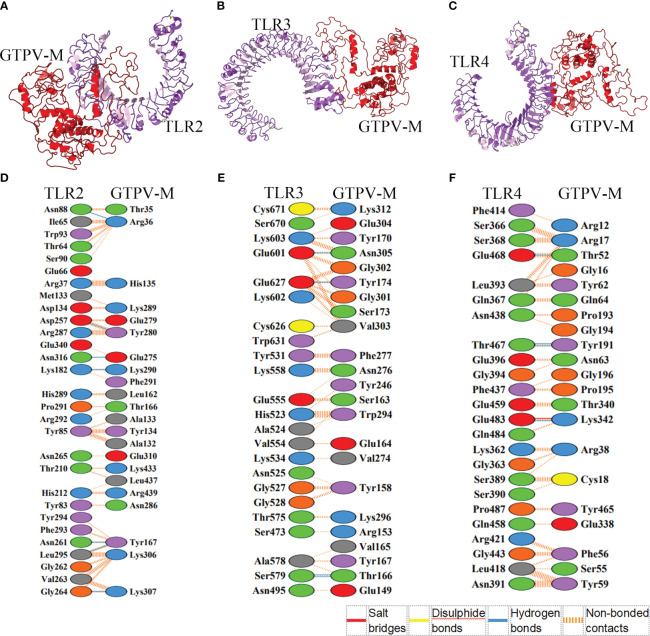
Molecular docking between GTPV-M vaccine and TLR2, TLR3 and TLR4. **(A–C)**. The GTPV-M vaccine was docked to TLR2, TLR3, and TLR4, respectively. The purple indicates TLR2, TLR3, and TLR4, respectively while the red indicates the vaccine construct; **(D–F)**. The interacting residues in TLR2-GTPV-M (7 hydrogen bonds and 260 non-bonded contacts), TLR3-GTPV-M (4 hydrogen bonds and 196 non-bonded contacts), and TLR4-GTPV-M (1 salt bridge, 4 hydrogen bonds and 127 non-bonded contacts) complex, respectively.

The HawkDock web server was used to dock the constructed GTPV-M vaccine to human immune receptor TLR2, TLR3 and TLR4, respectively. For each docking, top 100 models were achieved based on docking scores. The docking scores and binding free energies of the optimal model of TLR2-GTPV-M (score: -7270.23; binding free energy: -27.25 kcal/mol, **Figure 4A**), TLR3-GTPV-M (score: -5384.65; binding free energy: -39.84 kcal/mol, **Figure 4B**) and TLR4-GTPV-M complex (score: -6208.05; binding free energy: -59.42 kcal/mol, **Figure 4C**) were all very low, indicating that GTPV-M vaccine has a strong binding affinity with TLR2, TLR3, and TLR4, respectively.

The optimal models of vaccine-TLR complex were visualized and analyzed by PDBsum. Analysis of the binding interface of TLR2-GTPV-M complex revealed 7 hydrogen bonds and 260 non-bonded contacts. In addition, 22 interfacial residues of GTPV-M were found, while the corresponding TLR2 had 29 interfacial residues (**Figure 4D**). Interaction analysis of TLR3-GTPV-M complex showed 4 hydrogen bonds and 196 non-bonded contacts. TLR3-GTPV-M contained 23 interfacial residues, and the corresponding TLR3 had 23 interfacial residues (**Figure 4E**). Interaction analysis of TLR4-GTPV-M complex showed 1 salt bridge, 4 hydrogen bonds and 127 non-bonded contacts. GTPV-M had 21 interfacial residues, while the corresponding TLR4 had 24 interfacial residues (**Figure 4F**). Overall, there were 7, 4, and 5 sites of action in TLR2-GTPV-M, TLR3-GTPV-M, and TLR4-GTPV-M models, respectively, indicating a strong interaction between the GTPV-M vaccine and TLR2, TLR3, TLR4.

### 3.7 Molecular dynamics stimulation

**Figure 5:**
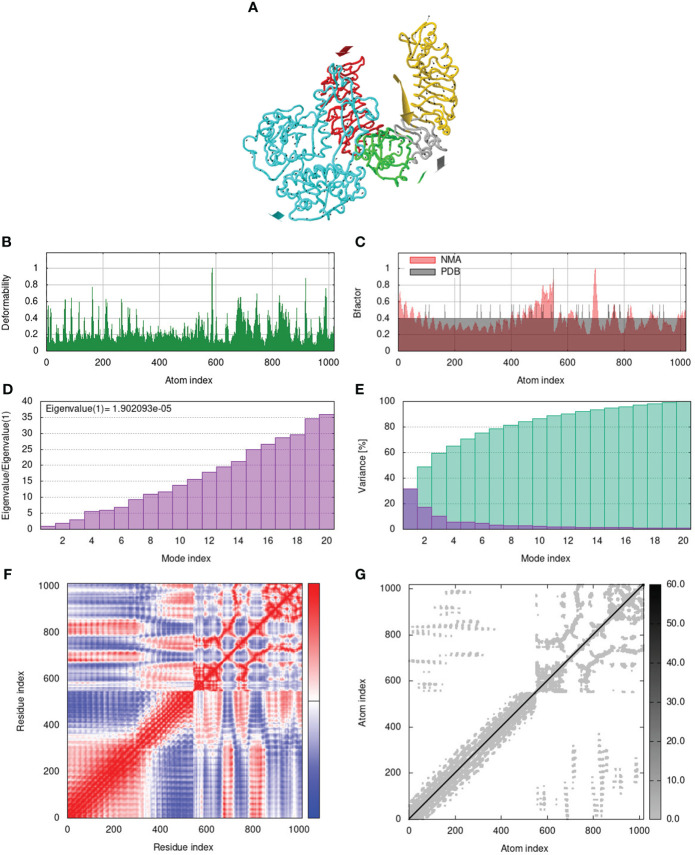
Molecular dynamics simulation of GTPV-M-TLR2 complex. **(A)**. Mobility of GTPV-M-TLR2 complex; **(B)**. Deformability plot; **(C)**. B-factor plot; **(D)**. Eigenvalues plot; **(E)**. Structural variance plot. Individual (purple) and cumulative (green) variances are shown as colored bars; **(F)**. Covariance plot. Matrix shows correlated (red), non-correlated (white), and anti-correlated (blue) motions of paired residues; **(G)**. Elastic network model. Darker grey indicates stiffer regions.

**Figure 6:**
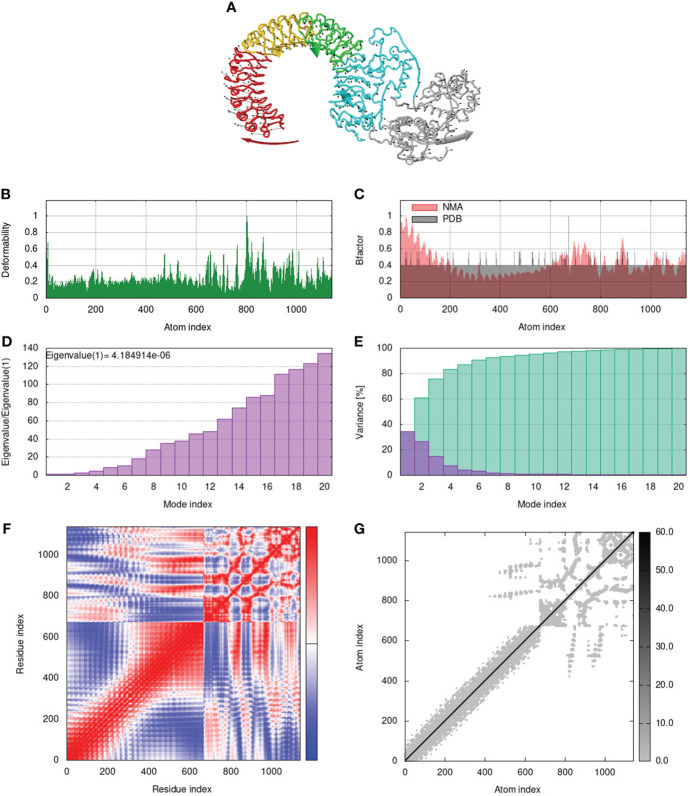
Molecular dynamics simulation of GTPV-M-TLR3 complex. **(A)**. Mobility of GTPV-M-TLR3 complex; **(B)**. Deformability plot; **(C)**. B-factor plot; **(D)**. Eigenvalues plot; **(E)**. Structural variance plot. Individual (purple) and cumulative (green) variances are shown as colored bars; **(F)**. Covariance plot. Matrix shows correlated (red), non-correlated (white), and anti-correlated (blue) motions of paired residues; **(G)**. Elastic network model. Darker grey indicates stiffer regions.

**Figure 7:**
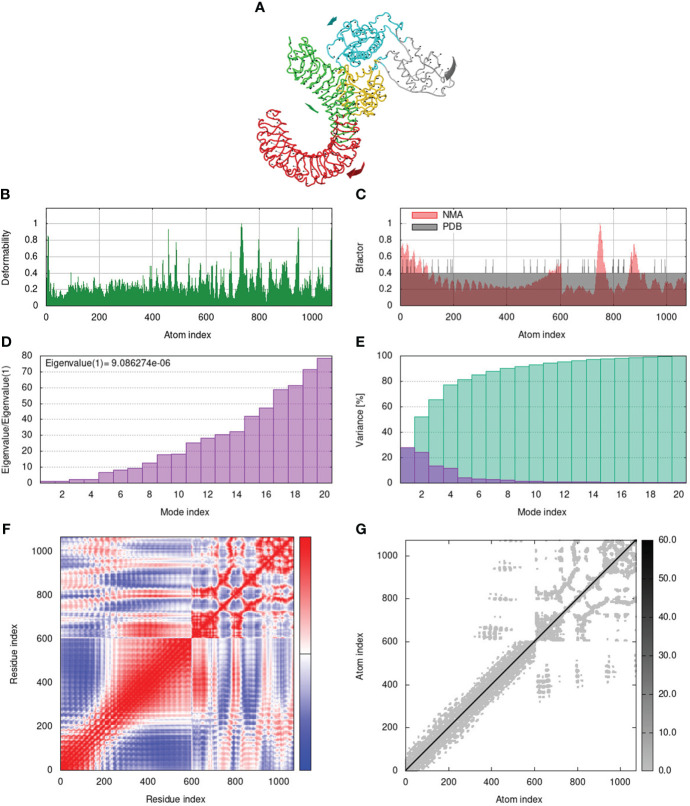
Molecular dynamics simulation of GTPV-M-TLR4 complex. **(A)**. Mobility of GTPV-M-TLR4 complex; **(B)**. Deformability plot; **(C)**. B-factor plot; **(D)**. Eigenvalues plot; **(E)**. Structural variance plot. Individual (purple) and cumulative (green) variances are shown as colored bars; **(F)**. Covariance plot. Matrix shows correlated (red), non-correlated (white), and anti-correlated (blue) motions of paired residues; **(G)**. Elastic network model. Darker grey indicates stiffer regions.

To assess the stability and movement of docked vaccine-TLR complexes, molecular dynamics stimulation via iMODS web server was performed. The mobility of TLR2-GTPV-M, TLR3-GTPV-M, and TLR4-GTPV-M complex is shown in **Figures 5A**, **6A**, **7A**, respectively. The deformability plots show that there is minimal distortion in GTPV-M-TLR2/3/4 complex, respectively (**Figures 5B**, **6B**, **7B**). B-factor plots illustrate the relationship between the mobility of the docked composite NMA and the PDB score (representing the mean RMSD) (**Figures 5C**, **6C**, **7C**). The eigenvalues of TLR2-GTPV-M, TLR3-GTPV-M and TLR4-GTPV-M complex are 1.902093e-05 (**Figure 5D**), 4.184914e-06 (**Figure 6D**) and 9.086274e-06 (**Figure 7D**), respectively, which indicates GTPV-M-TLR complexes are stable. Variance plots are associated with each normal mode representing the complex of individual variance (purple) and cumulative variance (green) (**Figures 5E**, **6E**, **7E**). Covariance plots are used to characterize the motions of correlated (red), non-correlated (white), or anti-correlated (blue) atoms in the dynamic regions of the complex molecules (**Figures 5F**, **6F**, **7F**). The elastic network model of vaccine-TLRs complexes studies the stiffness of TLR2-GTPV-M, TLR3-GTPV-M and TLR4-GTPV-M complex, respectively. Darker grey indicates stiffer regions while lighter dots indicate flexible regions (**Figures 5G**, **6G**, **7G**).

### 3.8 Immune simulation of GTPV-M vaccine

**Figure 8:**
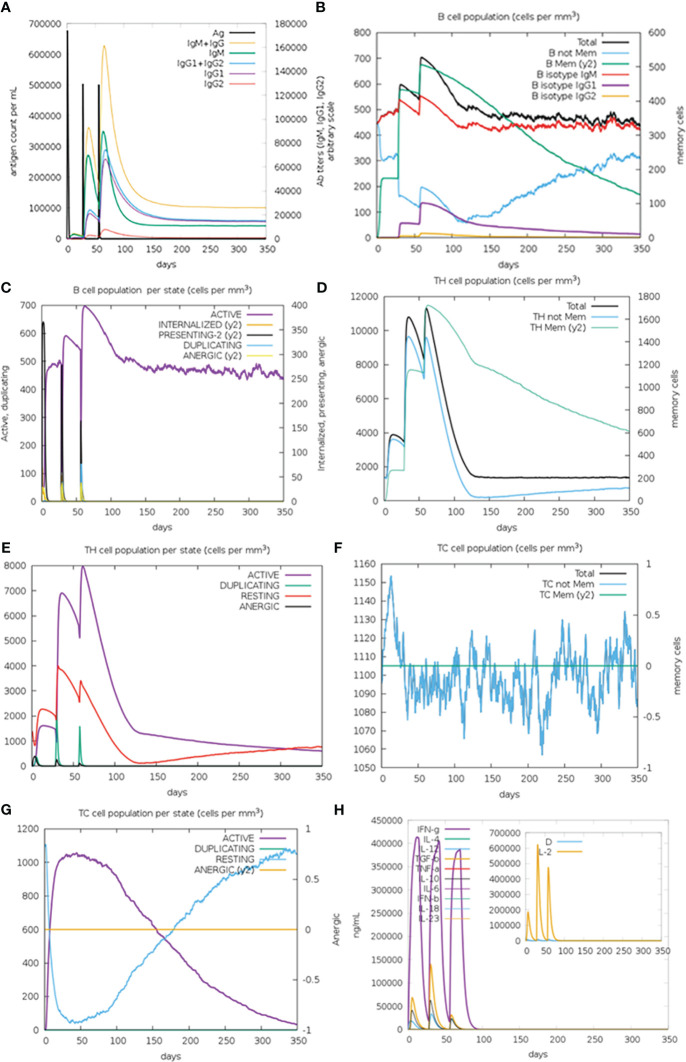
Immune simulation of GTPV-M vaccine. **(A)**. Immunoglobulin response against GTPV-M vaccine; **(B)**. B cell population; **(C)**. B cell population per state; **(D)**. TH cell population; **(E)**. TH cell population per state; **(F)**. TC cell population; **(G)**. TC cell population per state; **(H)**. Concentration of interleukins and cytokines. In the illustration, the letter D is a warning sign.

In silico immune simulation results demonstrated that after the second and third administration of GTPV-M vaccine, there was a remarkable increase in antibody titers of IgG1+IgG2, IgM and IgM+IgG (**Figure 8A**). There was also obvious increase in the number of B isotype (IgM) and memory B cells following the second and the third injection of GTPV-M vaccine (**Figure 8B**). Meanwhile, active B cells were significantly induced during the vaccination period (**Figure 8C**). Total helper T (TH) cells and active TH cells were also remarkably induced by vaccination during the simulation time (**Figures 8D, E**). Besides, cytotoxic T (TC) cells were observed during the immune simulation, with the maximum number of TC cells exceeding 1,150 cells per mm^3^ (**Figure 8F**). The number of activated cytotoxic T (TC) cells was significantly increased after chimeric antigen injection while the number of resting TC cells was decreased sharply (**Figure 8G**). Additionally, the constructed GTPV-M vaccine can induce significantly higher levels of IFN-γ as well as IL-2 after every injection (**Figure 8H**). These data show that GTPV-M vaccine was predicted to induce a robust immune response against GTPV.

### 3.9 Codon optimization and in silico cloning

**Figure 9:**
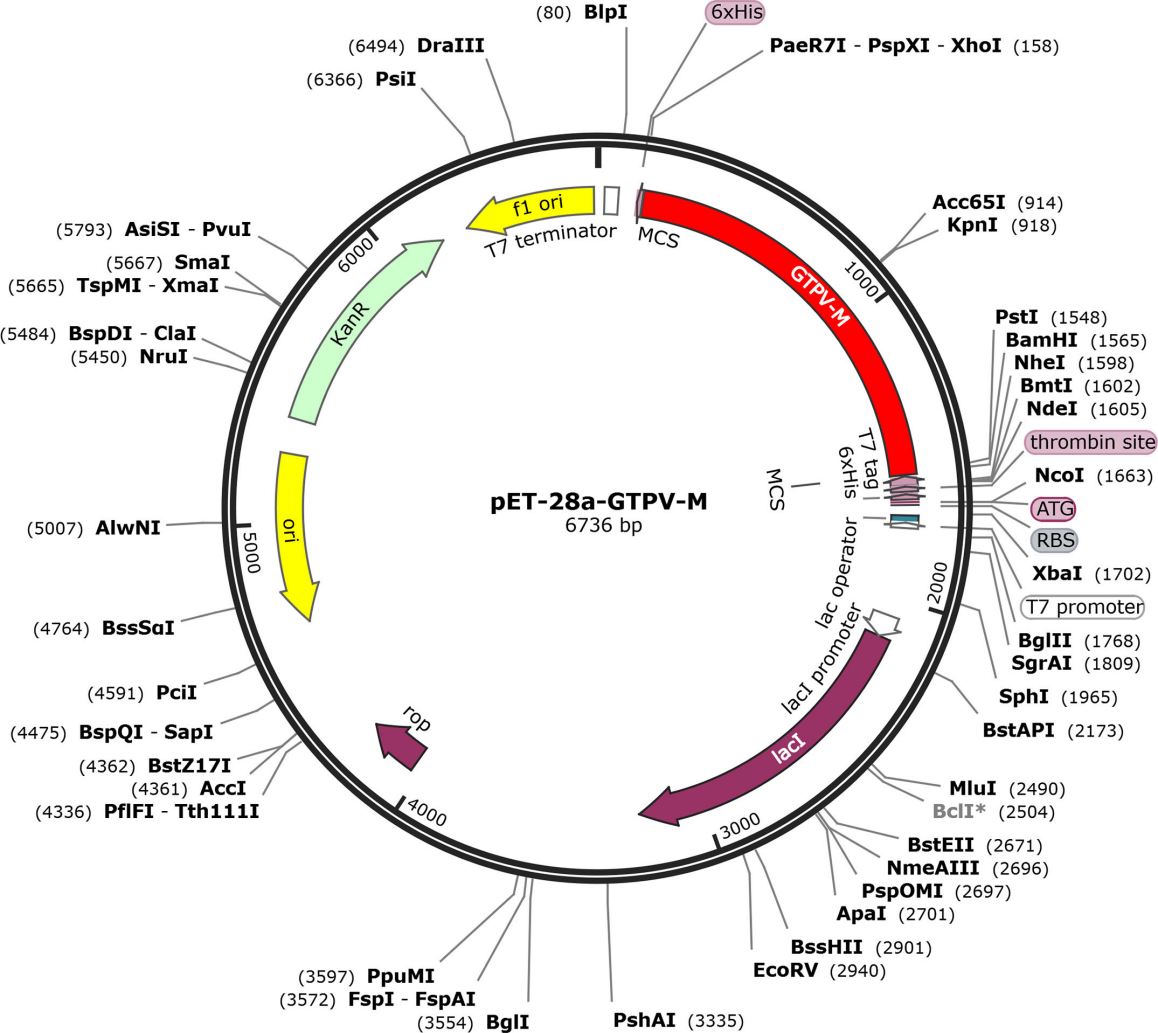
In silico cloning of GTPV-M vaccine into pET-28a (+) expression vector.

The GTPV-M vaccine construct was subjected to reverse-translation and codon optimization using the JCat online tool(**Supplementary Table 1**). The evaluated CAI value was 1.0 and the GC content was 44.68% for the improved DNA sequence, suggesting that the designed vaccine has a promising potential for stable and high expression in the *E.coli* expression system. Finally, the optimized DNA sequence of the GTPV-M vaccine was cloned into the pET-28a (+) vector with *Bam*HI and *Xho*I restriction sites to construct a recombinant plasmid using SnapGene software (**Figure 9**).

## 4 Discussion

Currently, vaccination is most effective against viral diseases (Plotkin, 2014). Conventional vaccines are not only costly, but also require a long time and do not have a high success rate. A multi-epitope vaccine can effectively avoid these disadvantages of conventional vaccines by combining reverse vaccinology with immunoinformatics approaches. It predicts the dominant B-cell antigen epitopes and T-cell antigen epitopes in the pathogen-coding amino acid sequence by computer, and then designs a multi-epitope vaccine with multiple immunodominant antigen epitopes (Ayyagari et al., 2022; Huang et al., 2022; Ullah et al., 2023; Waqas et al., 2023a; Waqas et al., 2023b).

In this study, a multi-epitope vaccine against GTPV was constructed. We first screened dominant CTL, HTL, and B-cell epitopes which were desired to induce a strong immune response. The linkers AAY, GPGPG, and KK play important roles in generating extended conformations, protein folding, and functional domain segregation, resulting in a more stable protein structure that enhances the immunogenicity of the multi-epitope vaccine antigen. They ensure that each epitope individually triggers an immune response and prevent new epitopes from appearing and compromising vaccine efficacy (Shamriz et al., 2016). Therefore, the screened CTL, HTL, and B- cell epitopes were constructed into a multi-epitope vaccine using AAY, GPGPG, and KK ligation, respectively. An adjuvant β-defensin was incorporated into the vaccine’s N-terminal using the EAAAK linker to enhance immunogenicity based on previous research (Adyns et al., 2023). The vaccine construct comprises 467 amino acids, exhibiting good antigenicity and solubility, without allergenicity or toxicity. The secondary structure of GTPV-M consisted of α-helix (48.18%), extended strands (19.27%), random coils (25.05%), and β-turns (7.49%). Naturally unfolded protein regions and convoluted helical motifs are referred to as “structural antigens”. An increase in these two structures may help to recognize antibodies produced after infection (Jiang et al., 2023). The vaccine’s 3D structure was predicted and further refined. Our results show that 86.45% of the amino acid residues of GTPV-M are located in the predominant region, which suggests that the predictive quality of the refined model is acceptable.

Toll-like receptors (TLRs) are pattern recognition receptors (PRRs) that are expressed on various cell types, including innate immune and non-immune cells. They play a central role in innate immunity through the recognition of conserved pathogen-associated molecular patterns (PAMPs) derived from a variety of microorganisms (Kawasaki and Kawai, 2014). Previous studies have shown that the activation of innate immunity and the production of antiviral responses can be initiated by TLR2 and TLR4 (Jyotisha et al., 2022). Stable contacts between the multi-epitope vaccine and TLR2 and TLR4 were verified by molecular docking. TLR3 can mediate the transcriptional induction of type I interferon (IFN), pro-inflammatory cytokines and chemokines, which collectively establish an antiviral host response (Chen et al., 2021). Molecular docking results show that GTPV-M can act in a stabilizing manner with TLR3. This determines the vaccine’s ability to elicit a stable immune response. Immune simulation results suggest that the constructed multi-epitope vaccine may trigger large amounts of IgG, IgM, and cytokines and stimulate the proliferation and differentiation of B and T cells into effector cells. Together, GTPV-M vaccine can elicit robust humoral and cellular immune responses, making it a promising vaccine candidate against GTPV. GTPV and SPPV were found to be antigenically similar, the constructed multi-epitope vaccine based on three highly conserved proteins among capripoxviruses are presumed to provide hosts effective cross-protection against both GTPV and SPPV.

Nevertheless, the present study has certain limitations. The designed vaccine is assumed to be immunogenic based on various immunoinformatics techniques. However, the accuracy of these approaches is not perfect, and it remains uncertain how effectively the simulated vaccine will protect hosts against GTPV infection. The safety and protective efficacy of the designed GTPV multi-epitope subunit vaccine will be assessed and compared with that of existing live attenuated vaccines through *in vitro* and *in vivo* experiments in the next phase.

## 5 Conclusion

A multi-epitope vaccine named GTPV-M, was successfully constructed based on reverse vaccinology and immunoinformatics approaches. GTPV-M vaccine consists of an adjuvant β-defensin, 12 CTL epitopes, 3 HTL epitopes and 12 B-cell epitopes, which has good solubility, immunogenicity, antigenicity, no sensitization or cytotoxicity. The vaccine is able to stabilize the interaction with TLR2, TLR3, and TLR4, and can induce a strong and long-lasting immune response in the host. The construction of GTPV multi-epitope vaccine provides a new solution for the prevention and control of goatpox.

## Data availability statement

The datasets presented in this study can be found in online repositories. The names of the repository/repositories and accession number(s) can be found in the article/**Supplementary Material**.

## Author contributions

QL: Software, Visualization, Writing – review & editing. MW: Methodology, Writing – review & editing. YW: Methodology, Writing – review & editing. FP: Conceptualization, Funding acquisition, Visualization, Writing – original draft, Writing – review & editing.

## References

[B1] Adyns L. Proost P. Struyf S. (2023). Role of defensins in tumor biology. Int. J. Mol. Sci. 24 (6), 5268. doi: 10.3390/ijms24065268 36982340 PMC10049535

[B2] Ahmad S. Navid A. Farid R. Abbas G. Ahmad F. Zaman N.. (2020). Design of a novel multi Epitope-Based vaccine for pandemic coronavirus disease (COVID-19) by vaccinomics and probable prevention strategy against avenging zoonotics. Eur. J. Pharm. Sci. 151, 105387. doi: 10.1016/j.ejps.2020.105387 32454128 PMC7245302

[B3] Aiman S. Alhamhoom Y. Ali F. Rahman N. Rastrelli L. Khan A.. (2022). Multi-epitope chimeric vaccine design against emerging Monkeypox virus *via* reverse vaccinology techniques- a bioinformatics and immunoinformatics approach. Front. Immunol. 13. doi: 10.3389/fimmu.2022.985450 PMC9452969 36091024

[B4] Andreatta M. Nielsen M. (2016). Gapped sequence alignment using artificial neural networks: Application to the MHC class I system. Bioinformatics. 32 (4), 511–517. doi: 10.1093/bioinformatics/btv639 26515819 PMC6402319

[B5] Ayyagari V. S. Venkateswarulu T. C. Abraham Peele K. Srirama K. (2022). Design of a multi-epitope-based vaccine targeting M-protein of SARS-CoV2: An immunoinformatics approach. J. Biomol. Struct. Dyn. 40 (7), 2963–2977. doi: 10.1080/07391102.2020.1850357 33252008 PMC7754933

[B6] Aziz S. Almajhdi F. N. Waqas M. Ullah I. Salim M. A. Khan N. A.. (2022). Contriving multi-epitope vaccine ensemble for monkeypox disease using an immunoinformatics approach. Front. Immunol. 13. doi: 10.3389/fimmu.2022.1004804 PMC9606759 36311762

[B7] Bhanot V. Balamurugan V. Bhanuprakash V. Venkatesan G. Sen A. Yadav V.. (2009). Expression of P32 protein of goatpox virus in Pichia pastoris and its potential use as a diagnostic antigen in ELISA. J. Virol. Methods 162 (1-2), 251–257. doi: 10.1016/j.jviromet.2009.08.020 19733197

[B8] Bhanuprakash V. Hosamani M. Venkatesan G. Balamurugan V. Yogisharadhya R. Singh R. K. (2012). Animal poxvirus vaccines: A comprehensive review. Expert Rev. Vaccines 11 (11), 1355–1374. doi: 10.1586/erv.12.116 23249235

[B9] Bhanuprakash V. Hosamani M. Venkatesan G. Singh R. K. (2022). Long-term protective immunity to goatpox in goats after a single immunization with a live attenuated goatpox vaccine. Arch. Virol. 167 (10), 2035–2040. doi: 10.1007/s00705-022-05505-8 35752986

[B10] Bienert S. Waterhouse A. de Beer T. A. Tauriello G. Studer G. Bordoli L.. (2017). The SWISS-MODEL Repository-new features and functionality. Nucleic Acids Res. 45 (D1), D313–D319. doi: 10.1093/nar/gkw1132 27899672 PMC5210589

[B11] Bisht H. Weisberg A. S. Moss B. (2008). Vaccinia virus l1 protein is required for cell entry and membrane fusion. J. Virol. 82 (17), 8687–8694. doi: 10.1128/JVI.00852-08 18596103 PMC2519644

[B12] Buchan D. Jones D. T. (2019). The PSIPRED Protein Analysis Workbench: 20 years on. Nucleic Acids Res. 47 (W1), W402–W407. doi: 10.1093/nar/gkz297 31251384 PMC6602445

[B13] Calis J. J. Maybeno M. Greenbaum J. A. Weiskopf D. De Silva A. D. Sette A.. (2013). Properties of MHC class I presented peptides that enhance immunogenicity. PloS Comput. Biol. 9 (10), e1003266. doi: 10.1371/journal.pcbi.1003266 24204222 PMC3808449

[B14] Chen Y. Lin J. Zhao Y. Ma X. Yi H. (2021). Toll-like receptor 3 (TLR3) regulation mechanisms and roles in antiviral innate immune responses. J. Zhejiang Univ Sci. B. 22 (8), 609–632. doi: 10.1631/jzus.B2000808 34414698 PMC8377577

[B15] Davies D. H. McCausland M. M. Valdez C. Huynh D. Hernandez J. E. Mu Y.. (2005). Vaccinia virus H3L envelope protein is a major target of neutralizing antibodies in humans and elicits protection against lethal challenge in mice. J. Virol. 79 (18), 11724–11733. doi: 10.1128/JVI.79.18.11724-11733.2005 16140750 PMC1212608

[B16] Dhanda S. K. Vir P. Raghava G. P. (2013). Designing of interferon-gamma inducing MHC class-II binders. Biol. Direct. 8, 30. doi: 10.1186/1745-6150-8-30 24304645 PMC4235049

[B17] Dharanesha N. K. Khorajiya J. H. Shivaraj B. M. Saminathan M. Byregowda S. M. (2020). Clinicopathological and molecular investigation of sheep pox in southern districts of Karnataka, India. Indian J. Veterinary Pathology. 44 (1), 43. doi: 10.5958/0973-970X.2020.00009.7

[B18] Dong R. Chu Z. Yu F. Zha Y. (2020). Contriving Multi-Epitope subunit of vaccine for COVID-19: Immunoinformatics approaches. Front. Immunol. 11. doi: 10.3389/fimmu.2020.01784 PMC7399176 32849643

[B19] Doytchinova I. A. Flower D. R. (2007). VaxiJen: A server for prediction of protective antigens, tumour antigens and subunit vaccines. BMC Bioinf. 8, 4. doi: 10.1186/1471-2105-8-4 PMC1780059 17207271

[B20] Grote A. Hiller K. Scheer M. Munch R. Nortemann B. Hempel D. C.. (2005). JCat: A novel tool to adapt codon usage of a target gene to its potential expression host. Nucleic Acids Res. 33 (Web Server issue), W526–W531. doi: 10.1093/nar/gki376 15980527 PMC1160137

[B21] Gupta S. Kapoor P. Chaudhary K. Gautam A. Kumar R. Raghava G. P. (2013). In silico approach for predicting toxicity of peptides and proteins. PloS One 8 (9), e73957. doi: 10.1371/journal.pone.0073957 24058508 PMC3772798

[B22] Hamdi J. Munyanduki H. Omari T. K. El H. M. Fassi F. O. (2021). Capripoxvirus infections in ruminants: A review. Microorganisms 9 (5), 902. doi: 10.3390/microorganisms9050902 33922409 PMC8145859

[B23] Hebditch M. Carballo-Amador M. A. Charonis S. Curtis R. Warwicker J. (2017). Protein-Sol: A web tool for predicting protein solubility from sequence. Bioinformatics 33 (19), 3098–3100. doi: 10.1093/bioinformatics/btx345 28575391 PMC5870856

[B24] Heo L. Lee H. Seok C. (2016). GalaxyRefineComplex: Refinement of protein-protein complex model structures driven by interface repacking. Sci. Rep. 6, 32153. doi: 10.1038/srep32153 27535582 PMC4989233

[B25] Hooper J. W. Custer D. M. Schmaljohn C. S. Schmaljohn A. L. (2000). DNA vaccination with vaccinia virus L1R and A33R genes protects mice against a lethal poxvirus challenge. Virology 266 (2), 329–339. doi: 10.1006/viro.1999.0096 10639319

[B26] Hooper J. W. Custer D. M. Thompson E. (2003). Four-gene-combination DNA vaccine protects mice against a lethal vaccinia virus challenge and elicits appropriate antibody responses in nonhuman primates. Virology 306 (1), 181–195. doi: 10.1016/s0042-6822(02)00038-7 12620810 PMC9628742

[B27] Hosamani M. Nandi S. Mondal B. Singh R. K. Rasool T. J. Bandyopadhyay S. K. (2004). A Vero cell-attenuated Goatpox virus provides protection against virulent virus challenge. Acta Virol. 48 (1), 15–21. 15230470

[B28] Hou W. Wu H. Wang S. Wang W. Wang B. Wang H. (2023). Designing a multi-epitope vaccine to control porcine epidemic diarrhea virus infection using immunoinformatics approaches. Front. Microbiol. 14. doi: 10.3389/fmicb.2023.1264612 PMC10538973 37779715

[B29] Huang S. Zhang C. Li J. Dai Z. Huang J. Deng F.. (2022). Designing a multi-epitope vaccine against coxsackievirus B based on immunoinformatics approaches. Front. Immunol. 13. doi: 10.3389/fimmu.2022.933594 PMC9682020 36439191

[B30] Jensen K. K. Andreatta M. Marcatili P. Buus S. Greenbaum J. A. Yan Z.. (2018). Improved methods for predicting peptide binding affinity to MHC class II molecules. Immunology 154 (3), 394–406. doi: 10.1111/imm.12889 29315598 PMC6002223

[B31] Jiang F. Liu Y. Xue Y. Cheng P. Wang J. Lian J.. (2023). Developing a multiepitope vaccine for the prevention of SARS-CoV-2 and monkeypox virus co-infection: A reverse vaccinology analysis. Int. Immunopharmacol. 115, 109728. doi: 10.1016/j.intimp.2023.109728 36652758 PMC9832108

[B32] Jyotisha Singh S. Qureshi I. A. (2022). Multi-epitope vaccine against SARS-CoV-2 applying immunoinformatics and molecular dynamics simulation approaches. J. Biomol. Struct. Dyn. 40 (7), 2917–2933. doi: 10.1080/07391102.2020.1844060 33164664 PMC7682209

[B33] Karki M. Kumar A. Venkatesan G. Arya S. Pandey A. B. (2018). Genetic analysis of L1R myristoylated protein of Capripoxviruses reveals structural homogeneity among poxviruses. Infect. Genet. Evol. 58, 224–231. doi: 10.1016/j.meegid.2018.01.001 29306003

[B34] Kawasaki T. Kawai T. (2014). Toll-like receptor signaling pathways. Front. Immunol. 5. doi: 10.3389/fimmu.2014.00461 PMC4174766 25309543

[B35] Kelley L. A. Mezulis S. Yates C. M. Wass M. N. Sternberg M. J. (2015). The Phyre2 web portal for protein modeling, prediction and analysis. Nat. Protoc. 10 (6), 845–858. doi: 10.1038/nprot.2015.053 25950237 PMC5298202

[B36] Ko J. Park H. Heo L. Seok C. (2012). GalaxyWEB server for protein structure prediction and refinement. Nucleic Acids Res. 40 (Web Server issue), W294–W297. doi: 10.1093/nar/gks493 22649060 PMC3394311

[B37] Kushwaha A. Kumar A. Madhavan A. Goswami D. Venkatesan G. Poulinlu G. (2019). Immunogenic proteins of capripox virus: Potential applications in Diagnostic/Prophylactic developments. Hosts Viruses 6 (6), 130–140. doi: 10.17582/journal.hv/2019/6.6.130.140

[B38] Laskowski R. A. Jablonska J. Pravda L. Varekova R. S. Thornton J. M. (2018). PDBsum: Structural summaries of PDB entries. Protein Sci. 27 (1), 129–134. doi: 10.1002/pro.3289 28875543 PMC5734310

[B39] Lopez-Blanco J. R. Aliaga J. I. Quintana-Orti E. S. Chacon P. (2014). IMODS: Internal coordinates normal mode analysis server. Nucleic Acids Res. 42 (Web Server issue), W271–W276. doi: 10.1093/nar/gku339 24771341 PMC4086069

[B40] Lopez-Blanco J. R. Garzon J. I. Chacon P. (2011). IMod: Multipurpose normal mode analysis in internal coordinates. Bioinformatics. 27 (20), 2843–2850. doi: 10.1093/bioinformatics/btr497 21873636

[B41] Madhavan A. Venkatesan G. Kumar A. Arya S. Pandey A. B. (2020). Comparative sequence and structural analysis of the ORF095 gene, a vaccinia virus A4L homolog of capripoxvirus in sheep and goats. Arch. Virol. 165 (6), 1419–1431. doi: 10.1007/s00705-020-04623-5 32307603

[B42] Magnan C. N. Zeller M. Kayala M. A. Vigil A. Randall A. Felgner P. L.. (2010). High-throughput prediction of protein antigenicity using protein microarray data. Bioinformatics 26 (23), 2936–2943. doi: 10.1093/bioinformatics/btq551 20934990 PMC2982151

[B43] Moin A. T. Patil R. B. Tabassum T. Araf Y. Ullah M. A. Snigdha H. J.. (2022). Immunoinformatics approach to design novel subunit vaccine against the Epstein-Barr virus. Microbiol. Spectr. 10 (5), e115122. doi: 10.1128/spectrum.01151-22 PMC9603631 36094198

[B44] Pham T. H. Rahaman N. Lila M. Lai H. Nguyen L. T. Van Nguyen G.. (2021). Molecular phylogenetics of a recently isolated goat pox virus from Vietnam. BMC Vet. Res. 17 (1), 115. doi: 10.1186/s12917-021-02777-1 33685458 PMC7938542

[B45] Plotkin S. (2014). History of vaccination. Proc. Natl. Acad. Sci. U. S. A. 111 (34), 12283–12287. doi: 10.1073/pnas.1400472111 25136134 PMC4151719

[B46] Puigbo P. Romeu A. Garcia-Vallve S. (2008). HEG-DB: A database of predicted highly expressed genes in prokaryotic complete genomes under translational selection. Nucleic Acids Res. 36 (Database issue), D524–D527. doi: 10.1093/nar/gkm831 17933767 PMC2238906

[B47] Rao T. V. Bandyopadhyay S. K. (2000). A comprehensive review of goat pox and sheep pox and their diagnosis. Anim. Health Res. Rev. 1 (2), 127–136. doi: 10.1017/s1466252300000116 11708598

[B48] Rapin N. Lund O. Bernaschi M. Castiglione F. (2010). Computational immunology meets bioinformatics: The use of prediction tools for molecular binding in the simulation of the immune system. PloS One 5 (4), e9862. doi: 10.1371/journal.pone.0009862 20419125 PMC2855701

[B49] Rcheulishvili N. Mao J. Papukashvili D. Liu C. Wang Z. Zhao J.. (2023). Designing multi-epitope mRNA construct as a universal influenza vaccine candidate for future epidemic/pandemic preparedness. Int. J. Biol. Macromol. 226, 885–899. doi: 10.1016/j.ijbiomac.2022.12.066 36521707

[B50] Saha S. Raghava G. P. (2006). Prediction of continuous B-cell epitopes in an antigen using recurrent neural network. Proteins. 65 (1), 40–48. doi: 10.1002/prot.21078 16894596

[B51] Saha S. Raghava G. P. (2007). Prediction methods for B-cell epitopes. Methods Mol. Biol. 409, 387–394. doi: 10.1007/978-1-60327-118-9_29 18450017

[B52] Santhamani R. Yogisharadhya R. Venkatesan G. Shivachandra S. B. Pandey A. B. Ramakrishnan M. A. (2014). Molecular characterization of Indian sheeppox and goatpox viruses based on RPO30 and GPCR genes. Virus Genes 49 (2), 286–291. doi: 10.1007/s11262-014-1095-3 24952423

[B53] Shamriz S. Ofoghi H. Moazami N. (2016). Effect of linker length and residues on the structure and stability of a fusion protein with malaria vaccine application. Comput. Biol. Med. 76, 24–29. doi: 10.1016/j.compbiomed.2016.06.015 27393958

[B54] Sippl M. J. (1993). Recognition of errors in three-dimensional structures of proteins. Proteins 17 (4), 355–362. doi: 10.1002/prot.340170404 8108378

[B55] Suleman M. Khan S. H. Rashid F. Khan A. Hussain Z. Zaman N.. (2023). Designing a multi-epitopes subunit vaccine against human herpes virus 6A based on molecular dynamics and immune stimulation. Int. J. Biol. Macromol. 244, 125068. doi: 10.1016/j.ijbiomac.2023.125068 37245745

[B56] Sumana K. Revanaiah Y. Shivachandra S. B. Mothay D. Apsana R. Saminathan M.. (2020). Molecular phylogeny of Capripoxviruses based on major immunodominant protein (P32) reveals circulation of host specific sheeppox and goatpox viruses in small ruminants of India. Infect. Genet. Evol. 85, 104472. doi: 10.1016/j.meegid.2020.104472 32711078

[B57] Tadesse B. Hamid M. Hamid A. (2022). Transmission dynamics and economic impacts of sheeppox and goatpox disease outbreak in Chifra district of Afar Region Ethiopia. Heliyon. 8 (6), e9674. doi: 10.1016/j.heliyon.2022.e09674 PMC9194592 35711991

[B58] Tian H. Chen Y. Wu J. Shang Y. Liu X. (2010). Serodiagnosis of sheeppox and goatpox using an indirect ELISA based on synthetic peptide targeting for the major antigen P32. Virol. J. 7, 245. doi: 10.1186/1743-422X-7-245 20854693 PMC2949846

[B59] Tulman E. R. Afonso C. L. Lu Z. Zsak L. Sur J. H. Sandybaev N. T.. (2002). The genomes of sheeppox and goatpox viruses. J. Virol. 76 (12), 6054–6061. doi: 10.1128/jvi.76.12.6054-6061.2002 12021338 PMC136203

[B60] Tuppurainen E. Venter E. H. Shisler J. L. Gari G. Mekonnen G. A. Juleff N.. (2017). Review: Capripoxvirus diseases: Current status and opportunities for control. Transbound Emerg. Dis. 64 (3), 729–745. doi: 10.1111/tbed.12444 26564428 PMC5434826

[B61] Ullah A. Waqas M. Aziz S. Rahman S. U. Khan S. Khalid A.. (2023). Bioinformatics and immunoinformatics approach to develop potent multi-peptide vaccine for coxsackievirus B3 capable of eliciting cellular and humoral immune response. Int. J. Biol. Macromol. 239, 124320. doi: 10.1016/j.ijbiomac.2023.124320 37004935

[B62] Venkatesan G. Kumar T. M. Sankar M. Kumar A. Dashprakash M. Arya S.. (2018). Expression and evaluation of recombinant P32 protein based ELISA for sero-diagnostic potential of capripox in sheep and goats. Mol. Cell Probes. 37, 48–54. doi: 10.1016/j.mcp.2017.11.005 29158139

[B63] Waqas M. Aziz S. Bushra A. Halim S. A. Ali A. Ullah S.. (2023a). Employing an immunoinformatics approach revealed potent multi-epitope based subunit vaccine for lymphocytic choriomeningitis virus. J. Infect. Public Health 16 (2), 214–232. doi: 10.1016/j.jiph.2022.12.023 36603375

[B64] Waqas M. Aziz S. Lio P. Khan Y. Ali A. Iqbal A.. (2023b). Immunoinformatics design of multivalent epitope vaccine against monkeypox virus and its variants using membrane-bound, enveloped, and extracellular proteins as targets. Front. Immunol. 14. doi: 10.3389/fimmu.2023.1091941 PMC9908764 36776835

[B65] Wass M. N. Kelley L. A. Sternberg M. J. (2010). 3DLigandSite: Predicting ligand-binding sites using similar structures. Nucleic Acids Res. 38 (Web Server issue), W469–W473. doi: 10.1093/nar/gkq406 20513649 PMC2896164

[B66] Waterhouse A. Bertoni M. Bienert S. Studer G. Tauriello G. Gumienny R.. (2018). SWISS-MODEL: Homology modelling of protein structures and complexes. Nucleic Acids Res. 46 (W1), W296–W303. doi: 10.1093/nar/gky427 29788355 PMC6030848

[B67] Weng G. Wang E. Wang Z. Liu H. Zhu F. Li D.. (2019). HawkDock: A web server to predict and analyze the protein-protein complex based on computational docking and MM/GBSA. Nucleic Acids Res. 47 (W1), W322–W330. doi: 10.1093/nar/gkz397 31106357 PMC6602443

[B68] Wiederstein M. Sippl M. J. (2007). ProSA-web: Interactive web service for the recognition of errors in three-dimensional structures of proteins. Nucleic Acids Res. 35 (Web Server issue), W407–W410. doi: 10.1093/nar/gkm290 17517781 PMC1933241

[B69] Wilkins M. R. Gasteiger E. Bairoch A. Sanchez J. C. Williams K. L. Appel R. D.. (1999). Protein identification and analysis tools in the ExPASy server. Methods Mol. Biol. 112, 531–552. doi: 10.1385/1-59259-584-7:531 10027275

[B70] Zeng X. Chi X. Li W. Hao W. Li M. Huang X.. (2014). Complete genome sequence analysis of goatpox virus isolated from China shows high variation. Vet. Microbiol. 173 (1-2), 38–49. doi: 10.1016/j.vetmic.2014.07.013 25113672

[B71] Zewdie G. Derese G. Getachew B. Belay H. Akalu M. (2021). Review of sheep and goat pox disease: Current updates on epidemiology, diagnosis, prevention and control measures in Ethiopia. Anim. Dis. 1 (1), 28. doi: 10.1186/s44149-021-00028-2 34806086 PMC8591591

[B72] Zheng M. Jin N. Liu Q. Huo X. Li Y. Hu B.. (2009). Immunogenicity and protective efficacy of Semliki forest virus replicon-based DNA vaccines encoding goatpox virus structural proteins. Virology 391 (1), 33–43. doi: 10.1016/j.virol.2009.05.031 19559453

[B73] Zhou T. Jia H. Chen G. He X. Fang Y. Wang X.. (2012). Phylogenetic analysis of Chinese sheeppox and goatpox virus isolates. Virol. J. 9, 25. doi: 10.1186/1743-422X-9-25 22264255 PMC3398307

[B74] Zhu Y. Li Y. Bai B. Fang J. Zhang K. Yin X.. (2018). Construction of an attenuated goatpox virus AV41 strain by deleting the TK gene and ORF8-18. Antiviral Res. 157, 111–119. doi: 10.1016/j.antiviral.2018.07.008 30030019

