# Supplementary material for: Design of a multi-epitope vaccine against goatpox virus using an immunoinformatics approach
Source: Front Cell Infect Microbiol. 2024 Feb 29;13:1309096. doi: 10.3389/fcimb.2023.1309096 (PMC10937444; doi:10.3389/fcimb.2023.1309096)

Supplementary Table 1. Condon optimization of the constructed GTPV multi-epitope vaccine.

| Vaccine construct | Protein sequence | Condon optimization (E.coli K12) |
| --- | --- | --- |
| GTPV multi-epitope vaccine | GIINTLQKYYCRVRGGRCAVLSCLPKEEQIGKCSTRGRKCCRRKKEAAAKSTSLSFEMYAAYNQENNNFMYAAYISDVVPELKAAYPSQSSGYGYAAYISPSQSSGYAAYILSMVFLYYAAYLLTPDQKAYAAYHTYDFESYYAAYLSEVTYRFYAAYISSLSEVTYAAYFLSYKEVNYAAYCSIQEKLGYGPGPGSAYVIRLSSAIKIINGPGPGTLSAYVIRLSSAIKIGPGPGVVILSMVFLYYVKKMKKVGKWMAHRFPDFSYYVKKGGVENFTEYFSGLCNAKKKFLIWEKVEKSGGVENKKANEMKNGIWNRVGKWMKKNEKISSKLEQTAEATSKKDQKAYVPGLMTAALNIKKHWTSYLDTFFSNTPTIKKATETYDLLTPDQKAYVKKDIEIGSIVFRQNKGCNKKNTVVKDFETYVKQKCTKKVFLLDRMNLFDKIISDKKNVTNYHTYDFESYYST | GGTATCATCAACACCCTGCAGAAATACTACTGCCGTGTTCGTGGTGGTCGTTGCGCTGTTCTGTCTTGCCTGCCGAAAGAAGAACAGATCGGTAAATGCTCTACCCGTGGTCGTAAATGCTGCCGTCGTAAAAAAGAAGCTGCTGCTAAATCTACCTCTCTGTCTTTCGAAATGTACGCTGCTTACAACCAGGAAAACAACAACTTCATGTACGCTGCTTACATCTCTGACGTTGTTCCGGAACTGAAAGCTGCTTACCCGTCTCAGTCTTCTGGTTACGGTTACGCTGCTTACATCTCTCCGTCTCAGTCTTCTGGTTACGCTGCTTACATCCTGTCTATGGTTTTCCTGTACTACGCTGCTTACCTGCTGACCCCGGACCAGAAAGCTTACGCTGCTTACCACACCTACGACTTCGAATCTTACTACGCTGCTTACCTGTCTGAAGTTACCTACCGTTTCTACGCTGCTTACATCTCTTCTCTGTCTGAAGTTACCTACGCTGCTTACTTCCTGTCTTACAAAGAAGTTAACTACGCTGCTTACTGCTCTATCCAGGAAAAACTGGGTTACGGTCCGGGTCCGGGTTCTGCTTACGTTATCCGTCTGTCTTCTGCTATCAAAATCATCAACGGTCCGGGTCCGGGTACCCTGTCTGCTTACGTTATCCGTCTGTCTTCTGCTATCAAAATCGGTCCGGGTCCGGGTGTTGTTATCCTGTCTATGGTTTTCCTGTACTACGTTAAAAAAATGAAAAAAGTTGGTAAATGGATGGCTCACCGTTTCCCGGACTTCTCTTACTACGTTAAAAAAGGTGGTGTTGAAAACTTCACCGAATACTTCTCTGGTCTGTGCAACGCTAAAAAAAAATTCCTGATCTGGGAAAAAGTTGAAAAATCTGGTGGTGTTGAAAACAAAAAAGCTAACGAAATGAAAAACGGTATCTGGAACCGTGTTGGTAAATGGATGAAAAAAAACGAAAAAATCTCTTCTAAACTGGAACAGACCGCTGAAGCTACCTCTAAAAAAGACCAGAAAGCTTACGTTCCGGGTCTGATGACCGCTGCTCTGAACATCAAAAAACACTGGACCTCTTACCTGGACACCTTCTTCTCTAACACCCCGACCATCAAAAAAGCTACCGAAACCTACGACCTGCTGACCCCGGACCAGAAAGCTTACGTTAAAAAAGACATCGAAATCGGTTCTATCGTTTTCCGTCAGAACAAAGGTTGCAACAAAAAAAACACCGTTGTTAAAGACTTCGAAACCTACGTTAAACAGAAATGCACCAAAAAAGTTTTCCTGCTGGACCGTATGAACCTGTTCGACAAAATCATCTCTGACAAAAAAAACGTTACCAACTACCACACCTACGACTTCGAATCTTACTACTCTACC |

Supplementary Figure 1. The secondary structure of the constructed GTPV multi-epitope vaccine.


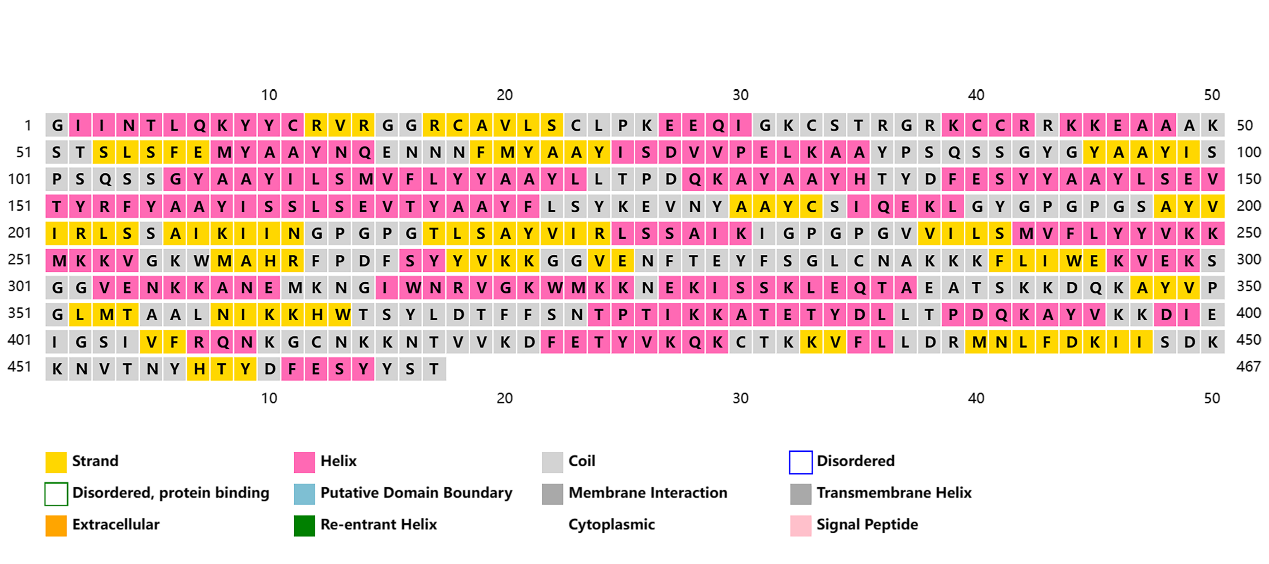

Supplement: Supplementary file 1 [file DataSheet_1.docx]
